# Incidence and prognostic value of tumour cells detected by RT–PCR in peripheral blood stem cell collections from patients with Ewing tumour

**DOI:** 10.1038/sj.bjc.6603438

**Published:** 2006-10-31

**Authors:** J Vermeulen, S Ballet, O Oberlin, M Peter, G Pierron, E Longavenne, V Laurence, J Kanold, P Chastagner, O Lejars, J-Y Blay, P Marec-Berard, J Michon, O Delattre, G Schleiermacher

**Affiliations:** 1 Département d'Oncologie Pédiatrique, Institut Curie, Paris, France; 2 Unité de Génétique Somatique, Institut Curie, Paris, France; 3 Département d'Oncologie Pédiatrique, Institut Gustave Roussy, Villejuif, France; 4 Département d'Oncologie Médicale, Institut Curie, Paris, France; 5 Centre de Cancérologie Pédiatrique, INSERM CIC 501, Clermont-Ferrand, France; 6 Département d'Oncologie Pédiatrique, Hôpital d'Enfants, Nancy, France; 7 Département d'Oncologie Pédiatrique, Hôpital Clocheville, Tours, France; 8 Département d'Oncologie Médicale, Hôpital Edouard-Herriot, Lyon, France; 9 Département d'Oncologie Pédiatrique, Centre Léon Bérard, Lyon, France; 10 INSERM U509, Institut Curie, Paris, France

**Keywords:** ewing tumour, PBSC, tumour cell contamination, RT–PCR, outcome

## Abstract

To retrospectively evaluate the incidence of tumour cell contamination of peripheral blood stem cell (PBSC) collections and to correlate these data with the clinical outcome after high-dose chemotherapy (HDCT) with stem cell rescue in patients with a high-risk Ewing tumour. Peripheral blood stem cell collections obtained from 171 patients were analysed. Tumour contamination was assessed by reverse transcriptase–polymerase chain reaction (RT–PCR). The files of 88 patients who underwent HDCT followed by PBSC reinfusion were reviewed in detail, and their outcome compared to the PBSC RT–PCR results. Seven of 88 PBSC collections (8%) contained tumour cells as detected by RT–PCR. Peripheral blood stem cells were collected after a median of five cycles of chemotherapy. No clinical factor predictive of tumour cell contamination of PBSC harvest could be identified. Event-free survival (EFS) and overall survival (OS) of the whole study population were 45.3 % and 51.8 % at 3 years from the date of the graft, respectively. Forty-five patients relapsed with a median time of 15 months after graft, only four of whom had tumour cell contamination of the PBSC harvest. Tumour cell contamination of PBSC collection is rare and does not seem to be associated with a significantly poorer EFS or OS in this high-risk population.

Ewing tumours (ET) represent the second most common primary bone malignancy and account for 4% of childhood and adolescent malignancies. They are neoplasms with aggressive local and metastatic behaviour, most often presenting as bone or less frequently as soft tissue masses.

These tumours are characterised by a specific tumour cell marker consisting of a balanced chromosomal translocation involving chromosome 22 and chromosome 11, or less frequently, chromosome 21, 2, 7, or 17. These balanced translocations lead to the fusion of the *EWS* gene on chromosome 22 to members of the ETS family of transcription factor genes *FLI*, *ERG*, or *FEV* in approximately 87%, 10%, and less than 1% of tumours, or *ETV1* or *E1AF* in rare cases, respectively (Turc-Carel *et al*, 1983; Delattre *et al*, 1992; Zucman *et al*, 1993; Sorensen *et al*, 1994; Jeon *et al*, 1995; Kaneko *et al*, 1996; Peter *et al*, 1997; Kovar, 1998). These fusion genes are transcribed in tumour cells and resulting chimaeric transcripts can be detected by reverse transcriptase–polymerase chain reaction (RT–PCR). Whereas cytogenetic analysis is technically difficult and its results often obtained very late, the search of the ET-specific fusion transcript by RT–PCR is a rapid, specific, and sensitive (1/10^6^ cells) diagnostic test and can also be applied for the detection of minimal disease in peripheral blood (PBL) and bone marrow (BM) (Delattre *et al*, 1994).

In ET, prognostic factors include age, primary site, and tumour volume, the presence of metastases at diagnosis detected by conventional methods and histological response to induction chemotherapy (Cotterill *et al*, 2000; Paulussen *et al*, 2001; Jenkin *et al*, 2002). Bone marrow micrometastases and circulating tumour cells detected by RT–PCR were also recently reported as prognostic factors (Schleiermacher *et al*, 2003). Prognosis of ET remains poor, with 5-year relapse-free survival of 55 to 65% in patients with localised disease and not exceeding 25% in primary metastatic disease. Survival at 5 years after relapse is less than 20% (Cotterill *et al*, 2000; Paulussen *et al*, 2001; Jenkin *et al*, 2002).

Treatment of ET requires a combination of multi-agent chemotherapy because of the marked propensity for dissemination of the tumour and on the other hand surgery and/or radiotherapy to ensure the most effective local control. Several reports, mainly in children, have suggested that some patients with high-risk ET may benefit from high-dose chemotherapy (HDCT) followed by autologous stem cell rescue (Burdach *et al*, 1993; Ladenstein *et al*, 1995; Burdach and Jurgens, 2002). The patient's stem cells are usually harvested after induction chemotherapy when complete remission (CR) or very good partial remission (PR) is achieved.

Previous studies on small series of patients with ET have shown that tumour cells might contaminate peripheral sites, including autologous stem cell products from the patient (Toretsky *et al*, 1995; Leung *et al*, 1998; Fischmeister *et al*, 1999; Thomson *et al*, 1999; Montanaro *et al*, 1999; Yaniv *et al*, 2004). Data on the incidence and prognostic value of tumour cell contamination of peripheral blood stem cell (PBSC) collections have been controversial.

The purpose of this study was to retrospectively evaluate the incidence of tumour cell contamination of PBSC collections detected by RT–PCR in a large series of patients with a high-risk ET and to correlate these data with the clinical outcome after HDCT with stem cell rescue.

## PATIENTS AND METHODS

### Samples

The initial study cohort consisted of all patients treated for ET in French centres for whom a sample of a PBSC could be analysed at the laboratory between February 1992 and December 2003. These PBSC collections were obtained by CD34+ cell mobilisation with concomitant chemotherapy and granulocyte-stimulating factor (GCSF) or after priming with GCSF alone. All samples were received at the laboratory in less than 24 h following sampling. The search for the *EWS/FLI* or *EWS/ERG* fusion transcripts was performed by RT–PCR as described previously (Peter *et al*, 1995, 2001). In brief, RNA was isolated using the Trizole extraction kit, reverse transcribed using the GeneAmp RNA PCR kit and subjected to PCR in search of specific fusion transcript according to a previously described nested PCR amplification procedure between 1992 and 1998 and a real-time PCR procedure between 1999 and 2003 (Peter *et al*, 1995, 2001). The quality of RNA was controlled by a test amplification of the ubiquitously expressed *EWS* gene with identical PCR conditions, as published previously (Delattre *et al*, 1994; Fagnou *et al*, 1998; Peter *et al*, 2001). In case of PBSC collected over different days for the same patient, samples were analysed separately but one single positive sample was sufficient to consider positivity for the entire graft.

The search for tumour cells in PBSC samples was performed at time of harvesting and required no additional sampling. In no instance did the results influence therapeutic decisions. According to the French law at the time of the study, no informed consent was required for analysis of archived biologic material.

### Patients

The initial cohort comprised 171 patients with a high-risk ET for whom results of the search for an ET-specific fusion transcript in a PBSC sample were interpretable. Of these, 25 patients were not included in this study because they were enrolled in the randomised arms of an ongoing clinical trial (EuroEwing 99). Eighteen patients could not be included owing to a lack of sufficient clinical information. Another 28 patients could not be included because no molecular data of the tumour at diagnosis was available. Thus, for 100 patients clinical history was reviewed in detail. Twelve patients did not undergo HDCT, eight because of early progressive disease (PD), two because there was finally no indication, one because of cardiac insufficiency and the last one because he refused. For none of the non-included patients had a fusion transcript been found in PBSC collection.

Thus a total of 88 patients with a complete available data set for whom PBSC were analysed by RT–PCR and who underwent HDCT followed by PBSC reinfusion were included in this study. For these patients the diagnosis of ET was confirmed by the presence of an ET-specific fusion transcript in the primary tumour or in peripheral sites.

Most of the patients (*n*=71) were treated according to the French Society of Paediatric Oncology (SFOP) EW 88, or EW 93-97 protocols or according to the nonrandomised arms of the EuroEwing 99 protocol. These protocols include an induction chemotherapy consisting of a combination of Doxorubicin and Cyclophosphamide±Etoposide and Ifosphamide (EW 88/93-97), or Vincristine, Ifosphamide, Doxorubicin and Etoposide (EuroEwing 99). The other patients received induction chemotherapy according to protocols used for adult patients consisting essentially of various associations of the same drugs. Local treatment consisted of surgery and/or radiotherapy.

Peripheral blood stem cell were obtained by cytapheresis. For CD34+ cell mobilisation, conventional chemotherapy followed by GCSF treatment or GCSF alone in steady state was used. Conditioning treatment consisted of an association of Busulphan and Melphalan in 75 patients and other types of HDCT (Melphalan, Etoposide, Carboplatin, Ifosphamide, Thiotepa, and Cyclophosphamide) in the 13 remaining patients.

At diagnosis, metastatic disease was searched for by pulmonary CT scanner, technetium bone scintigraphy, and BM analysis. Disease status was also evaluated at time of stem cell collection, before HDCT and at the end of treatment. In case of search of tumour cells in PBL or BM samples by RT–PCR, the results were not known to clinicians and were not taken into account for treatment stratification.

### Statistical analysis

Correlation between clinical and molecular data was assessed by using the Fisher's exact test. Event-free survival (EFS) and overall survival (OS), indicated with the standard deviation, were estimated with the Kaplan–Meier method and compared by the log-rank test. A *P*-value of less than 0.05 was considered to be significant. Event-free survival was calculated from the day of PBSC reinfusion until the date of last follow-up or event (tumour progression or relapse). Overall survival was calculated from the day of PBSC reinfusion to the last follow-up or disease- or toxicity-related death.

## RESULTS

### Patients

The characteristics of the 88 patients with a high-risk ET who underwent HDCT are shown in Table 1.

The median age was 15 years (range 1–49 years). The incidence was higher in males than in females. Large tumours with a volume over 200 ml were observed in 36 of 71 evaluated patients (51%). The primary tumour origin was most frequently in bone (76 patients; 86%). Metastases were present at diagnosis in 32 patients (36%) with the lung being the most frequent site of metastatic disease.

**Figure 1:**
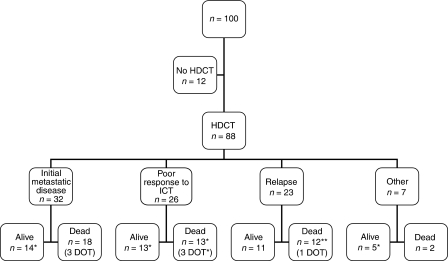
Indication of HDCT in 100 patients with a high-risk ET. HDCT: high-dose chemotherapy. ICT: induction chemotherapy. DOT: dead of toxicity. ^*^: Patients with tumour cell contamination of peripheral blood stem cell collection.

The indication for HDCT was metastatic disease at diagnosis in 32 patients (36%). Other indications for HDCT were poor response to induction chemotherapy defined as less than 90% of necrosis at surgery in 22 cases (25%) or clinical response less than 50% in case of inoperability in four cases (5%), or personal choice of the clinician in seven cases (8%). Finally, HDCT was indicated for treatment of relapse in 23 cases (26%) (Figure 1).

### Tumour cell contamination of peripheral blood stem cell collections

Only seven out of 88 PBSC harvests (8%) contained tumour cells as detected by RT–PCR. Clinical variables associated with tumour cell contamination of PBSC collection were searched for (Table 2). Neither the presence of metastases or more specifically BM metastases at diagnosis, nor the presence of circulating tumour cells or BM micrometastases determined by RT–PCR at diagnosis, nor the number of chemotherapy cycles before PBSC collection, nor the status of disease at the time of PBSC collection, nor the indication of HDCT (primary treatment or relapse) were significantly correlated with the presence of contaminating tumour cells in PBSC products. Likewise, no clinical factor predictive of tumour cell contamination of PBSC harvest could be found in the subgroup of patients with a primary indication of HDCT (*n*=65). The only patient with histologically diagnosed BM metastases at the time of harvesting also scored positive by RT–PCR.

Patient characteristics of the group of patients with tumour cell contamination of PBSC collections are shown in Table 3. In this group of patients, for five patients PBSC was collected during initial treatment whereas for the two others, PBSC was collected after metastatic relapse. At the time of harvesting, two patients were in CR, four in PR, and one in PD. Of the four patients in PR, two patients achieved CR by the time of HDCT.

Of the seven PBSC RT–PCR-positive patients, one patient died of complications immediately after HDCT, and four patients relapsed in metastatic sites within 1 month, 2, 20, and 30 months after stem cell rescue. Both patients whose indication of HDCT was relapse, relapsed again after graft. Of the four patients who relapsed, three have died and only one (patient 3) is still alive but not free of disease. The two other patients who did not relapse (patients 1 and 5) are alive in CR.

### Clinical outcome of the study population (*n*=88)

**Figure 2:**
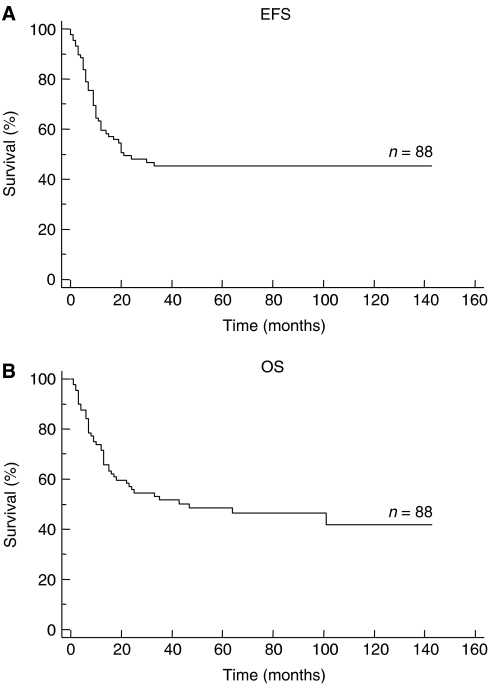
EFS (**A**) and OS (**B**) of 88 patients with a high-risk ET after stem cell rescue.

**Figure 3:**
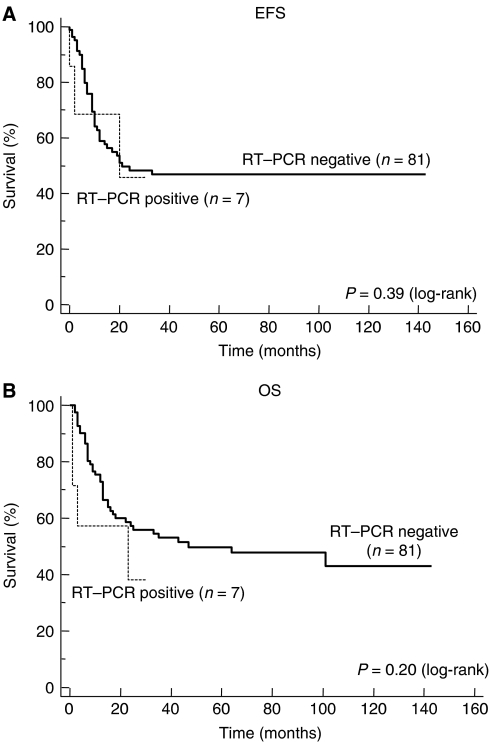
EFS (**A**) and OS (**B**) of patients with RT–PCR-negative and -positive harvest after stem cell rescue. In the RT–PCR-positive group, the latest event consisting of a relapse occurring at 30 months following HDCT has not been indicated intentionally.

The median follow-up, calculated by the inverse Kaplan–Meier method in order to take into account the real follow-up of all patients, was 69 months (range 1–143 months). Of the total group of 88 patients, 43 patients are alive, 38 in CR, one in SD, and four in PD. Event-free survival and OS of the whole study population are shown in Figure 2. They were 45.3% (±11%) and 51.8% (±11%) at 3 years from the date of the graft, respectively. Patients with tumour cell contamination of the PBSC collection did not present a statistically significant poorer EFS or OS (log-rank, *P*=0.39 and *P*=0.20, respectively; Figure 3). Likewise, no correlation between tumour cell contamination and poorer EFS or OS could be found in the subgroup of 65 patients with an indication of HDCT during primary treatment (log-rank, *P*=0.9 and *P*=0.72, respectively; figure not shown).

Of the 88 patients who underwent HDCT followed by autologous stem cell rescue, 45 patients relapsed with a median time of 15 months after graft (range <1–33 months) and of those 45 patients, 38 died of tumour recurrence. Whereas recurrences occurred purely locally in two out of the 41 patients with RT–PCR-negative harvest, no purely local recurrences were observed in the four patients with RT–PCR-positive harvest who relapsed (*P*=1.0; Table 4). Of the four patients with a positive harvest who relapsed, two relapsed in prior metastatic sites and the two others in a single different metastatic site.

## DISCUSSION

Tumour cell contamination in PBSC products that are reinfused after potentially curative HDCT has become an important issue in a number of haematological malignancies and solid tumours. In this study, we have analysed tumour cell contamination of PBSC collections in a large series of patients with ET and correlated these findings with clinical outcome.

Our data show that the incidence of tumour cell contamination of PBSC collections as determined by RT–PCR is very low (seven out of the 88 patients included in this study; 8%) in this group of patients with a high-risk ET. Taking into account all interpretable results of PBSC analysis by RT–PCR, the incidence was even lower (seven out of 171 cases; 4.1%), This finding is in contrast to the frequency of PBSC contamination observed in some previously published studies. In a recently published series all 11 patients had contaminating tumour cells in their PBSC harvests (Yaniv *et al*, 2004). Two other studies have also reported a high incidence of contamination by tumour cells, occurring in either marrow or PBSC grafts of all five patients in both studies (Toretsky *et al*, 1995; Leung *et al*, 1998). The incidence of tumour cell contamination of PBSC products in the present study (8%) is more consistent with the findings of three other studies which reported a proportion of patients with tumour cell contamination of PBSC samples of one out of 15 (6.7%), one out of nine (11.1%), and three out of 15 (20%), respectively (Fischmeister *et al*, 1999; Montanaro *et al*, 1999; Thomson *et al*, 1999). This important variability in incidence of tumour cell contamination in PBSC collections in previous reports might be due to different sensitivity and specificity of the techniques of detection, to different times of harvesting and to different disease status at the time of PBSC collection.

First, it is of concern that tumour cell detection by RT–PCR may yield false-positive and/or false-negative results. In our study, to avoid false-positive results generated by cross-contamination linked to the use of PCR amplification techniques, several strict precautions were taken. Samples were processed at different sites for RNA isolation, PCR amplification, and electrophoresis of PCR products and the two rounds of nested PCR amplification were processed without opening the tube (Peter *et al*, 1995). The observation that the type of fusion transcript was consistent between primary tumour and PBSC collection provided a good control for the specificity of the technique. Following the introduction of real-time PCR in 1999, the risk of carry-over contamination was even more reduced as no PCR products are manipulated with this method (Peter *et al*, 2001). Moreover since that time the GeneAmp RNA PCR kit has been used which contains an enzyme (Uracil N-glycosylase) in order to degrade specifically PCR products from previous PCR amplifications (Longo *et al*, 1990). To avoid false-negative results owing to an error during the procedure resulting in degraded RNAs, an internal control was established. The quality of RNA was controlled by a test amplification of the ubiquitously expressed *EWS* gene with identical PCR conditions. In the current study, a total of 34 patients were not included as no interpretable results of PBSC RT–PCR analysis could be obtained owing to poor RNA quality.

Second, the time of harvesting might influence the incidence of tumour cell contamination of PBSC collections. In a previous study, one patient had a positive harvest after two and three cycles of induction chemotherapy, whereas his harvest was negative after four cycles (Toretsky *et al*, 1995). More recently, another study reported that in two patients a first PBSC collection had had tumour contamination detected by RT–PCR but subsequently cleared after two additional cycles of chemotherapy (Thomson *et al*, 1999). In our study, all PBSC collections were performed after a median of five cycles of induction chemotherapy (a median of four cycles in the group of patients with and five cycles in the group of patients without tumour cell contamination of PBSC collection), most likely contributing to the observed low incidence of tumour cell contamination of PBSC collection. No positive samples were observed in the group of patients treated according to the EuroEwing 99 protocol (nonrandomised arms), which consists of a more intensive induction chemotherapy (six cycles of Vincristine, Ifosphamide, Doxorubicin, and Etoposide) in comparison to the previous EW 88 and EW 93–97 protocols. This could suggest that more intensive induction chemotherapy might eliminate circulating cells. However, owing to the small number of positive samples and because of the inclusion of only nonrandomised patients of the EuroEwing 99 protocol, the relevance of these findings with regard to current clinical practice needs to be interpreted with caution.

Third, the status of disease at the time of PBSC collection might also influence the incidence of tumour cell contamination. In our series, of the seven patients with positive harvest, only one patient (patient 7) had BM disease at the time of harvesting and all the patients with a negative harvest were free of disease in BM as detected histologically at that time. Unfortunately for none of the seven patients with tumour cell contamination of PBSC collection in this study, samples of BM for the detection of micrometastases at the time of PBSC collection could be studied. In a recently published study, six out of the six PBSC-positive patients tested had BM micrometastases at the time of harvesting (Yaniv *et al*, 2004). In two other studies tumour cells in BM detected by RT–PCR were observed in two out of the three PBSC-positive patients tested and in three out of the three PBSC-positive patients tested but also in the only PBSC-negative patient, respectively (Toretsky *et al*, 1995; Leung *et al*, 1998). Interestingly, in the two series with a low incidence of tumour cell contamination of PBSC collection, no cases of BM micometastases at the time of PBSC collection were observed in the first and only one case in the second study (Fischmeister *et al*, 1999; Thomson *et al*, 1999). This observation might suggest that micrometastatic disease in BM should be sought for before collecting PBSC.

Tumour cells present in autografts have been shown to be associated with poor outcome in patients with haematological malignancies or solid tumours. In a recently published study, the presence of occult tumour cells in aphaeresis products was correlated with worse EFS and OS in patients with high-risk primary breast cancer and with worse EFS in patients with metastatic breast cancer (Nieto *et al*, 2004). Another study concluded that breast cancer patients with more than three contaminating cells in their aphaeresis products represent a poor prognosis group (Syme *et al*, 2003). In malignant germ-cell tumours, the presence of contaminating tumour cells in PBSC harvests seems to predict a poor EFS and OS in patients undergoing HDCT and autologous PBSC reinfusion (Hildebrandt *et al*, 2000). As demonstrated in a recently published trial, myeloma patients with graft contamination of more than 4.5 × 10^5^ plasma cells kg^−1^ have a high risk of early disease progression after HDCT (Vogel *et al*, 2005).

To date, the prognostic significance of tumour cell contamination of PBSC collection from patients with ET has only been analysed in small series of patients. In a series of 11 patients, seven out of the 11 patients with contaminated PBSC harvest relapsed after graft and a possible correlation between the number of contaminating cells in the harvest and relapse after transplantation was suggested (Yaniv *et al*, 2004). In our study, no statistically significant difference in EFS or OS after graft could be found between patients with and without tumour cell contamination of PBSC collection. Owing to the low incidence of tumour cell contamination of PBSC collections, these results should be interpreted with caution. Furthermore, the prognostic significance of the contamination of PBSC collection might not be the same in patients with different clinical indications for HDCT. However, when the subgroup of patients with a primary indication of HDCT was analysed separately, no significant correlation could be found between tumour cell contamination of the PBSC collection and poorer EFS or OS.

The role of reinfused contaminating tumour cells for disease progression remains unclear. In our study, of the four patients with a positive harvest who relapsed, two patients relapsed in prior metastatic sites, indicating that remaining systemic disease might have been the primary cause of relapse rather than a direct effect of reinfusion of tumour cells. This suggests an insufficient cytoreductive capacity of HDCT rather than a direct effect of tumour cells contaminating the graft. Indeed, the benefit of HDCT has been questioned especially for patients with BM metastases (Oberlin *et al*, 2006). It might be possible that PBSC contamination reflects only a greater overall tumour burden, with reinfused cancer cells having no major role in disease relapse. Prior analyses in advanced breast cancer have suggested that contaminating tumour cells in PBSC collections may be a marker of widespread micrometastatic disease rather than a direct cause of recurrence after transplantation (Nieto *et al*, 2004). Furthermore, the biological significance of contaminating tumour cells in PBSC collection remains to be established. Indeed, two patients in our study did not relapse after graft and are still in CR, which might suggest that the autograft did not contain tumorigenic tumour cells with a metastatic potential. Several investigators have performed qualitative tests on micrometastatic tumour cells, including the determination of the viability and growth potential of these cells and the assessment of the invasive capacity in various solid tumours (Ross *et al*, 1993; Moss *et al*, 1994; Sharp *et al*, 1996). In ET, the oncogenic potential of tumour cells detected by RT–PCR in PBSC collection, although likely, remains to be proven.

In conclusion, our study shows that the incidence of tumour cell contamination of PBSC collection from patients with high-risk ET is low and that it does not seem to have an additional negative impact on outcome in this population already at risk.

## Figures and Tables

**Table 1:** Characteristics of patients with ET having undergone HDCT followed by autologous stem cell rescue

**Variable**	**Total (*n*=88)**
Age in years (median and range)	15 (1–49)
Sex ratio (male/female)	54/34
	
*Tumour volume (ml)*	
⩾200	36
<200	35
NE	17
	
*Primary tumour site*	
Bone	76
Head	5
Trunk	44
Limbs	27
Soft tissue	12
	
*Metastatic sites at diagnosis* (*n*=32)	
Pulmonary	7
Bone	7
Bone marrow	2
Mixed (pulmonary and/or bone and/or BM)	15
Other	1
	
*Treatment protocol*	
EW 88	4
EW 93-97	42
EE 99 (nonrandomised patients)	25
Other	17
	
Median number of chemotherapy cycles before PBSC collection	5 (2–10)
	
*Global disease status at PBSC collection*	
CR	37
PR	45
SD	3
PD	1
NE	2
	
*Conditioning regimen*	
Busulfan-Melphalan	75
Other	13

ET=Ewing tumour; HDCT=high-dose chemotherapy; BM=bone marrow; CR=complete remission; PR=partial remission; SD=stable disease; PD=progressive disease; NE=not evaluated; PBSC=peripheral blood stem cell.

**Table 2:** Clinical parameters of patients with RT–PCR-negative and -positive PBSC collection in the whole study population and in the patients with indication of HDCT during primary treatment

	**HDCT (all indications) (*n*=88)**		**HDCT (indication during primary treatment) (*n*=65)**		
	**RT–PCR PBSC collection**		**RT–PCR PBSC collection**		
**Variable**	**Negative (*n*=81)**	**Positive (*n*=7)**	**Negative (*n*=60)**	**Positive (*n*=5)**	***P*-value Fischer's exact test**
*Initial metastases*					
Yes	31	1	31	1	NS
No	50	6	29	4	
					
*Initial BM metastases*					
Yes	11	0	11	0	NS
No	70	7	49	5	
					
*Initial BM micrometastases*					
Yes	6	0	4	0	NS
No	20	1	19	1	
NE	55	6	37	4	
					
*Initial circulating tumour cells*					
Yes	2	1	1	1	NS
No	20	0	18	0	
NE	59	6	41	4	
					
*Number of chemotherapy cycles before PBSC collection (median)*					
⩽5 cycles	43	5	24	3	NS
>5 cycles	38	2	36	2	
					
*Metastases at the time of PBSC collection*					
Yes	33	3	19	1	NS
No	48	4	41	4	
					
*Disease status at the time of PBSC collection*					
CR	35	2	30	2	NS
No CR	44	5	30	3	
NE	2	0	0	0	
					
*Indication of HDCT*					
Primary treatment	60	5			NS
Relapse	21	2			

RT–PCR=reverse transcriptase–polymerase chain reaction; PBSC=peripheral blood stem cell; HDCT=high-dose chemotherapy; BM=bone marrow; CR=complete remission; NE=not evaluated; NS=not significant.

**Table 3:** Characteristics of patients with tumour cell contamination of PBSC collection detected by RT–PCR performed during primary treatment or following relapse

**Patient**	**Age**	**Sexe**	**Primary tumour**	**Tumour volume (ml)**	**Initial metastases**	**Indication of HDCT**	**Total cycles of chemotherapy before PBSC collection**	**Metastases at the time of PBSC collection**	**Disease status at the time of PBSC collection**	**Disease status before HDCT**	**HDCT**	**Relapse after HDCT (months)**	**Outcome (FU since diagnosis/since HDCT in months)**
1	15	F	Vertebra	NE	0	Macroscopic incomplete resection	5	0	PR	PR	MECA	/	CR (24/10)
2	13	M	Vertebra	<200	0	Poor response to ICT	8	0	CR	CR	Bu-Mel	/	DOT (8/1)
3	12	M	Fibula	<200	B (multiple)	Initial metastatic disease	4	B	PR	CR	Bu-Mel	30 (SC)	SD (38/30)
4	31	M	Rib	⩾200	0	Poor response to ICT	6	0	CR	CR	MEV	2 (D)	DOD (14/3)
5	20	M	Sacrum	<200	0	Poor response to ICT	3	0	PR	CR	Bu-Mel	/	CR (33/26)
6	12	F	Fibula	<200	0	Relapse (P)	2	P	PR	PR	Bu-Mel	20 (PV)	DOD (58/23)
7	14	M	Femur	⩾200	0	Relapse (B+BM)	2	B+BM	PD	PD	Paraplatin	<1 (B+BM+P)	DOD (9/1)

HDCT=high-dose chemotherapy during primary treatment (pt 1–5) or for relapse (pt 6–7); PBSC=peripheral blood stem cell; RT–PCR=reverse transcriptase–polymerase chain reaction; F=female; M=male; ICT=induction chemotherapy; P=pulmonary; B=bone; BM=bone marrow; PV=paravertebral; SC=subcutaneous; D=diaphragm; NE=not evaluated; PR=partial remission; CR=complete remission; PD=progressive disease; SD=stable disease; DOD=dead of disease; DOT=dead of toxicity; FU=follow-up; MECA=Melphalan, Cyclophosphamide, Paraplatin, Doxorubicin; Bu-Mel=Busulphan, Melphalan; MEV=Melphalan, Cyclophosphamide, Etoposide.

**Table 4:** Outcome after graft in 88 patients with ET

**Outcome after graft**	**Negative PBSC collection (*n*=81)**	**Positive PBSC collection (*n*=7)**
No relapse	40	3
Relapse	41	4
Median time (months)	16 (<1–33)	15 (<1–30)
Location		
Local	2	0
Metastatic	31	4
Both	8	0

ET, Ewing tumour; PBSC=peripheral blood stem cell.

## References

[bib1] Burdach S, Jurgens H (2002) High-dose chemoradiotherapy (HDC) in the Ewing family of tumors (EFT). Crit Rev Oncol Hematol 41: 169–189 11856593 10.1016/s1040-8428(01)00154-8

[bib2] Burdach S, Jurgens H, Peters C, Nurnberger W, Mauz-Korholz C, Korholz D, Paulussen M, Pape H, Dilloo D, Koscielniak E (1993) Myeloablative radiochemotherapy and hematopoietic stem-cell rescue in poor-prognosis Ewing's sarcoma. J Clin Oncol 11: 1482–1488 8101562 10.1200/JCO.1993.11.8.1482

[bib3] Cotterill SJ, Ahrens S, Paulussen M, Jurgens HF, Voute PA, Gadner H, Craft AW (2000) Prognostic factors in Ewing's tumor of bone: analysis of 975 patients from the European Intergroup Cooperative Ewing's Sarcoma Study Group. J Clin Oncol 18: 3108–3114 10963639 10.1200/JCO.2000.18.17.3108

[bib4] Delattre O, Zucman J, Melot T, Garau XS, Zucker JM, Lenoir GM, Ambros PF, Sheer D, Turc-Carel C, Triche TJ, Aurias A, Thomas G (1994) The Ewing family of tumors – a subgroup of small round-cell tumors defined by specific chimeric transcripts. N Engl J Med 331: 294–299 8022439 10.1056/NEJM199408043310503

[bib5] Delattre O, Zucman J, Plougastel B, Desmaze C, Melot T, Peter M, Kovar H, Joubert I, de Jong P, Rouleau G, Aurias A, Thomas G (1992) Gene fusion with an ETS DNA-binding domain caused by chromosome translocation in human tumours. Nature 359: 162–165 1522903 10.1038/359162a0

[bib6] Fagnou C, Michon J, Peter M, Bernoux A, Oberlin O, Zucker JM, Magdelenat H, Delattre O (1998) Presence of tumor cells in bone marrow but not in blood is associated with adverse prognosis in patients with Ewing's tumor. Societe Francaise d'Oncologie Pediatrique. J Clin Oncol 16: 1707–1711 9586882 10.1200/JCO.1998.16.5.1707

[bib7] Fischmeister G, Zoubek A, Jugovic D, Witt V, Ladenstein R, Fritsch G, Hocker P, Gadner H, Kovar H (1999) Low incidence of molecular evidence for tumour in PBPC harvests from patients with high-risk Ewing tumours. Bone Marrow Transplant 24: 405–409 10467330 10.1038/sj.bmt.1701924

[bib8] Hildebrandt M, Rick O, Salama A, Siegert W, Huhn D, Beyer J (2000) Detection of germ-cell tumor cells in peripheral blood progenitor cell harvests: impact on clinical outcome. Clin Cancer Res 6: 4641–4646 11156214

[bib9] Jenkin RD, Al-Fawaz I, Al-Shabanah M, Allam A, Ayas M, Khafaga Y, Memon M, Rifai S, Schultz H, Younge D (2002) Localised Ewing sarcoma/PNET of bone – Prognostic factors and international data comparison. Med Pediatr Oncol 39: 586–593 12376982 10.1002/mpo.10212

[bib10] Jeon IS, Davis JN, Braun BS, Sublett JE, Roussel MF, Denny CT, Shapiro DN (1995) A variant Ewing's sarcoma translocation (7;22) fuses the EWS gene to the ETS gene ETV1. Oncogene 10: 1229–1234 7700648

[bib11] Kaneko Y, Yoshida K, Handa M, Toyoda Y, Nishihira H, Tanaka Y, Sasaki Y, Ishida S, Higashino F, Fujinaga K (1996) Fusion of an ETS-family gene, E1AF, to EWS by t(17;22)(q12;q12) chromosome translocation in an undifferentiated sarcoma of infancy. Genes Chromosomes Cancer 15: 115–121 8834175 10.1002/(SICI)1098-2264(199602)15:2<115::AID-GCC6>3.0.CO;2-6

[bib12] Kovar H (1998) Ewing's sarcoma and peripheral primitive neuroectodermal tumors after their genetic union. Curr Opin Oncol 10: 334–342 9702401 10.1097/00001622-199807000-00010

[bib13] Ladenstein R, Gadner H, Hartmann O, Pico J, Biron P, Thierry P (1995) The European experience with megadose therapy and autologous bone marrow transplantation in solid tumors with poor prognosis : Ewing sarcoma, germ cell tumors and brain tumors. Wien Med Wochenschr 145: 55–57 7762255

[bib14] Leung W, Chen AR, Klann RC, Moss TJ, Davis JM, Noga SJ, Cohen KJ, Friedman AD, Small D, Schwartz CL, Borowitz MJ, Wharam MD, Paidas CN, Long CA, Karandish S, McMannis JD, Kastan MB, Civin CI (1998) Frequent detection of tumor cells in hematopoietic grafts in neuroblastoma and Ewing's sarcoma. Bone Marrow Transplant 222: 971–979 10.1038/sj.bmt.1701471 9849694

[bib15] Longo MC, Berninger MS, Hartley JL (1990) Use of uracil DNA glycosylase to control carry-over contamination in polymerase chain reactions. Gene 93(1): 125–128 2227421 10.1016/0378-1119(90)90145-h

[bib16] Montanaro L, Pession A, Trere D, Vici M, Prete A, Paolucci G, Derenzini M (1999) Detection of EWS chimeric transcripts by nested RT–PCR to allow reinfusion of uncontaminated peripheral blood stem cells in high-risk Ewing's tumor in childhood. Haematologica 84: 1012–1015 10553162

[bib17] Moss TJ, Cairo M, Santana VM, Weinthal J, Hurvitz C, Bostrom B (1994) Clonogenicity of circulating neuroblastoma cells: implications regarding peripheral blood stem cell transplantation. Blood 83: 3085–3089 7910052

[bib18] Nieto Y, Franklin WA, Jones RB, Berman SI, Pellom J, Baron AE, Shpall EJ (2004) Prognostic significance of occult tumor cells in the apheresis products of patients with advanced breast cancer receiving high-dose chemotherapy and autologous hematopoietic progenitor cell support. Biol Blood Marrow Transplant 10: 415–425 15148495 10.1016/j.bbmt.2004.02.004

[bib19] Oberlin O, Rey A, Desfachelles AS, Philip T, Plantaz D, Schmitt C, Plouvier E, Lejars O, Rubie H, Terrier P, Michon J (2006) Impact of high-dose busulfan plus melphalen as consolidation in metastatic ewing tumors: a study by the Société Française des Cancers de l'Enfant. J Clin Oncol 24: 3997–4002 16921053 10.1200/JCO.2006.05.7059

[bib20] Paulussen M, Ahrens S, Dunst J, Winkelmann W, Exner GU, Kotz R, Amann G, Dockhorn-Dworniczak B, Harms D, Muller-Weihrich S, Welte K, Kornhuber B, Janka-Schaub G, Gobel U, Treuner J, Voute PA, Zoubek A, Gadner H, Jurgens H (2001) Localized Ewing tumor of bone: final results of the Cooperative Ewing's Sarcoma Study CESS 86. J Clin Oncol 19: 1818–1829 11251014 10.1200/JCO.2001.19.6.1818

[bib21] Peter M, Couturier J, Pacquement H, Michon J, Thomas G, Magdelenat H, Delattre O (1997) A new member of the Ets family fused to EWS in Ewing tumors. Oncogene 14: 115–121 9121764 10.1038/sj.onc.1200933

[bib22] Peter M, Gilbert E, Delattre O (2001) A multiplex real-time PCR assay for the detection of gene fusions observed in solid tumors. Lab Invest 81: 905–912 11406651 10.1038/labinvest.3780299

[bib23] Peter M, Magdelenat H, Michon J, Melot T, Oberlin O, Zucker JM, Thomas G, Delattre O (1995) Sensitive detection of occult Ewing's cells by the reverse transcriptase-polymerase chain reaction. Br J Cancer 72: 96–100 7599072 10.1038/bjc.1995.283 PMC2034130

[bib24] Ross AA, Cooper BW, Lazarus HM, Mackay W, Moss TJ, Ciobanu N, Tallman MS, Kennedy MJ, Davidson NE, Sweet D, Winter C, Akard L, Jansen J, Copelan E, Meagher R, Herzig R, Klumpp T, Kahn D, Warner N (1993) Detection and viability of tumor cell collection from breast cancer patients using immunocytochemical and clonogenic assay techniques. Blood 82: 2605–2610 8219214

[bib25] Schleiermacher G, Peter M, Oberlin O, Philip T, Rubie H, Mechinaud F, Sommelet-Olive D, Landman-Parker J, Bours D, Michon J, Delattre O, Societe Francaise d'Oncologie Pediatrique (2003) Increased risk of systemic relapses associated with bone marrow micrometastasis and circulating tumor cell in localized Ewing tumor. J Clin Oncol 21: 85–91 12506175 10.1200/JCO.2003.03.006

[bib26] Sharp JG, Kessinger A, Mann S, Crouse DA, Armitage JO, Bierman P, Weisenburger DD (1996) Outcome of high-dose therapy and autologous transplantation in non-Hodgkin's lymphoma based on the presence of tumor in the marrow or infused hematopoietic harvest. J Clin Oncol 14: 214–219 8558200 10.1200/JCO.1996.14.1.214

[bib27] Sorensen PH, Lessnick SL, Lopez-Terrada D, Liu XF, Triche TJ, Denny CT (1994) A second Ewing's sarcoma translocation, t(21;22), fuses the EWS gene to another ETS-family transcription factor, ERG. Nat Genet 6: 146–151 8162068 10.1038/ng0294-146

[bib28] Syme R, Stewart D, Rodriguez-Galvez M, Luider J, Auer Y, Klassen J, Morris D, Brown C, Russell J, Gluck S (2003) Micrometastases in apheresis products predict shorter progression-free and overall survival in patients with breast cancer undergoing high-dose chemotherapy (HDCT) and autologous blood stem cell transplantation (ABSCT). Bone marrow Transplant 32: 307–311 12858203 10.1038/sj.bmt.1704133

[bib29] Thomson B, Hawkins D, Felgenhauer J, Radich J (1999) RT–PCR evaluation of peripheral blood, bone marrow and peripheral blood stem cells in children and adolescents undergoing VACIME chemotherapy for Ewing's sarcoma and alveolar rhabdomyosarcoma. Bone Marrow Transplant 24: 527–533 10482938 10.1038/sj.bmt.1701939

[bib30] Toretsky JA, Neckers L, Wexler LH (1995) Detection of (11;22)(q24;q12) translocation-bearing cells in peripheral blood progenitor cells of patients with ewing's sarcoma family of tumors. J Natl Cancer Inst 87: 385–386 7853420 10.1093/jnci/87.5.385

[bib31] Turc-Carel C, Philip I, Berger MP, Philip T, Lenoir G (1983) Chromosomal translocations in Ewing's sarcoma. N Engl J Med 309: 497–498

[bib32] Vogel W, Kopp HG, Kanz L, Einsele H (2005) Myeloma cell contamination of peripheral blood stem-cell grafts can predict the outcome in multiple myeloma patients after high-dose chemotherapy and autologous stem-cell transplatation. J Cancer Res Clin Oncol 131: 214–218 15616828 10.1007/s00432-004-0635-y PMC12161228

[bib33] Yaniv I, Cohen IJ, Stein J, Zilberstein J, Liberzon E, Atlas O, Grunshpan A, Sverdlov Y, Ash S, Zaizov R, Avigad S (2004) Tumor cells are present in stem cell harvests of ewings sarcoma patients and their persistence following transplantation is associated with relapse. Pediatr Blood Cancer 42: 404–409 15049010 10.1002/pbc.20022

[bib34] Zucman J, Melot T, Desmaze C, Ghysdael J, Plougastel B, Peter M, Zucker JM, Triche TJ, Sheer D, Turc-Carel C, Ambros P, Combaret V, Lenoir G, Aurias A, Thomas G, Delattre O (1993) Combinatorial generation of variable fusion proteins in the Ewing family of tumours. EMBO J 12: 4481–4487 8223458 10.1002/j.1460-2075.1993.tb06137.x PMC413872

